# Dengue virus serotype did not contribute to clinical severity or mortality in Taiwan’s largest dengue outbreak in 2015

**DOI:** 10.1186/s40001-023-01454-3

**Published:** 2023-11-06

**Authors:** Jih-Jin Tsai, Ko Chang, Chun-Hong Chen, Ching-Len Liao, Liang-Jen Chen, Yan-Yi Tsai, Ching-Yi Tsai, Ping-Chang Lin, Miao-Chen Hsu, Li-Teh Liu

**Affiliations:** 1 Tropical Medicine Center, Kaohsiung Medical University Hospital, Kaohsiung, Taiwan; 2 School of Medicine, College of Medicine, Kaohsiung Medical University, Kaohsiung, Taiwan; 3 Division of Infectious Diseases, Department of Internal Medicine, Kaohsiung Medical University Hospital, Kaohsiung, Taiwan; 4 Department of Internal Medicine, Kaohsiung Municipal Siaogang Hospital, Kaohsiung Medical University, Kaohsiung, Taiwan; 5 National Mosquito-Borne Diseases Control Research Center, National Health Research Institutes, Zhunan, Taiwan; 6 National Institute of Infectious Diseases and Vaccinology, National Health Research Institutes, Zhunan, Taiwan; 7 Department of Family Medicine, Pingtung Christian Hospital, Pingtung, Taiwan; 8 Department of Medical Laboratory Science and Biotechnology, College of Medical Technology, Chung Hwa University of Medical Technology, Tainan, Taiwan

**Keywords:** DENV-1, DENV-2, Dengue fever, Severe dengue, Mortality, Anti-dengue IgG

## Abstract

**Background:**

Dengue virus serotype 2 (DENV-2) was the major serotype in the 2015 dengue outbreak in Taiwan, while DENV-1 and DENV-3 were dominant between 2005 and 2014. We aimed to investigate whether DENV-2 contributed to disease severity and mortality in the outbreak in Kaohsiung city, Taiwan.

**Methods:**

We collected serum samples from dengue patients to detect the presence of DENV and determine the serotypes by using quantitative reverse transcription-polymerase chain reaction. Our cohorts comprised 105 DENV-1-infected cases and 1,550 DENV-2-infected cases. Demographic data, DENV serotype, and comorbidities were covariates for univariate and multivariate analyses to explore the association with severity and mortality.

**Results:**

The results suggested that DENV-1 persisted and circulated, while DENV-2 was dominant during the dengue outbreak that occurred between September and December 2015. However, DENV-2 did not directly contribute to either severity or mortality. Aged patients and patients with diabetes mellitus (DM) or moderate to severe chronic kidney disease (CKD) had a higher risk of developing severe dengue. The mortality of dengue patients was related to a higher Charlson comorbidity index score and severe dengue. Among DENV-2-infected patients and older patients, preexisting anti-dengue IgG, DM, and moderate to severe CKD were associated with severe dengue. Moreover, female sex and severe dengue were associated with a significantly higher risk of death.

**Conclusions:**

Our findings highlight the importance of timely serological testing in elderly patients to identify potential secondary infections and focus on the meticulous management of elderly patients with DM or moderate to severe CKD to reduce dengue-related death.

**Supplementary Information:**

The online version contains supplementary material available at 10.1186/s40001-023-01454-3.

## Introduction

Dengue virus (DENV) belongs to the genus Flavivirus and family Flaviviridae, and there are four different serotypes of DENV (DENV-1 to DENV-4) according to serological tests [1]. Infection by DENV in humans can result in a wide range of clinical consequences, including asymptomatic infections, dengue with warning signs, dengue without warning signs, severe dengue according to the 2009 World Health Organization (WHO) dengue guidelines [2] or undifferentiated fevers, dengue fever (DF), life-threatening dengue hemorrhagic fever (DHF) and dengue shock syndrome (DSS) according to the 1997 WHO dengue guidelines [3]. The impact of dengue on humans has increased over the past five decades due to frequent international travel, travel between urban and rural areas, population increases, and global warming, resulting in the widespread distribution of *Aedes* mosquitoes [4].

Dengue epidemics were recorded in the southern regions of Taiwan as early as the early twentieth century. Six major dengue outbreaks have occurred in Taiwan over the past four decades. The first dengue outbreak (dominant serotype DENV-2) occurred in 1981. Subsequently, dengue outbreaks occurred in 1988 (dominant serotype DENV-1), 2002 (dominant serotype DENV-2), 2007 (dominant serotype DENV-1), and 2014 (dominant serotype DENV-1), and the largest outbreak after World War II occurred in 2015 (dominant serotype DENV-2) [5]. The 2014 epidemic of the DENV-1 genotype I continued and resulted in sporadic cases in southern Taiwan between January and April 2015 [6–9]. The dengue outbreak in 2015 started in July in Tainan city. This DENV-2 cosmopolitan genotype outbreak spread from Tainan to its neighboring city, Kaohsiung, at the end of August [7]. This year's dengue outbreak was initiated by the importation of dengue cases. The results indicated that various factors may have been associated with this DF outbreak in Kaohsiung, such as higher monthly average temperatures, precipitation, consecutive rainfall, and elevated Breteau index. These factors may have increased mosquito breeding activity, thus aiding in the transmission of DENV [7]. In addition, a recent study by Pan et al. identified a possible relationship between the period of disease concealment and the number of imported dengue cases, which resulted in epidemics of indigenous dengue fever within local communities [10].

Previous studies suggested that DENV-1 and DENV-2 were the major DENV subtypes in the 2014 and 2015 outbreaks, respectively [5]. The mortality rate during the DF outbreak in 2015 (0.52%) was higher than that in 2014 (0.17%) [7]. It has been suggested that different serotypes of DENV are associated with different severities of dengue [11–13]. A recent meta-analysis study suggested that certain serotypes increased the risk of severe dengue with geographical effects [14]. The serotype effect on the disease severity as well as on the clinical outcomes in the 2015 dengue outbreak had not been investigated using the 2009 dengue guideline proposed by the WHO [2]. In addition, cocirculation of multiple serotypes of DENV during a single season increased the incidence of coinfections with more than one dengue serotype and was correlated with severe dengue [15]. Coinfection with multiple serotypes of DENV may result in the emergence of recombinant virus strains, which possibly increase disease severity [16] and the difficulty of vaccine development. Moreover, monitoring dengue serotype prevalence assists healthcare providers in predicting the potential severity of dengue cases and delivering suitable clinical care. In this study, we questioned whether serotypes other than DENV-2 were cocirculating and hypothesized that dengue serotype might play a role in clinical severity and outcomes. Therefore, we identified the dengue serotypes using serum samples collected between September and December 2015 and analyzed the association of DENV serotypes with dengue severity and mortality in Kaohsiung city during that period.

## Methods

### Ethics statement and sample collection

This research was approved by the Institutional Review Board of Kaohsiung Medical University Hospital (KMUH) (KMUHIRB-960195 and KMUHIRB-EXEMPT(II)-20160036). Hospitalized patients with dengue-like symptoms were invited to participate in this study, and their serum samples were collected between September and December 2015 at KMUH, Kaohsiung city, Taiwan. We obtained written informed consent before sample collection. All sera were collected between 0 and 22 days post symptom onset (PSO) using serum separation tubes (Becton Dickinson, USA), and days PSO ≤ 6 were regarded as acute-phase samples.

### Viral RNA extraction and qRT‒PCR

Viral RNA was extracted from 200 μL of serum sample using the PureLink Viral RNA Mini Kit (Life Technologies, USA) according to the instructions from the manufacturer and immediately subjected to real-time qRT‒PCR. Real-time qRT‒PCR was performed with a Brilliant II SYBR Green qRT‒PCR Low ROX Master Mix system (Agilent, USA). In brief, a 25 μL mixture containing 5 μL of sample RNA, 0.25 μM forward and reverse DENV detecting or molecular-typing primers each, 2 × Brilliant II SYBR Green qRT‒PCR Low ROX Master Mix, RT/RNase Block Enzyme Mixture and RNase-free water was assayed in an Mx3000P machine (Agilent, USA). Dengue-specific primers for DENV RNA detection were DN-F: CAA TAT GCT GAA ACG CGA GAG AAA and DN-R: CCC CAT CTA TTC AGA ATC CCT GCT. Serotype-specific primers for molecular serotyping were DN-F: CAA TAT GCT GAA ACG CGA GAG AAA, D1-R CGC TCC ATA CAT CTT GAA TGA G, D2-R: AAG ACA TTG ATG GCT TTT GA, D3-R: AAG ACG TAA ATA GCC CCC GAC and D4-R: AGG ACT CGC AAA AAC GTG ATG AAT. The criteria of a positive control were a threshold cycle (*C*_*t*_) value ≤ 30 and a Tm ≥ 79 °C, while a negative control had a *C*_*t*_ value ≥ 40 and a Tm < 79 °C. For the samples, a Ct value of ≤ 30 or a Tm ≥ 79 °C was considered positive [17].

### Determination of preexisting anti-dengue IgG by using InBios DENV detect IgG ELISA

We detected the presence of anti-DENV IgG in acute-phase sera by using the InBios DENV Detect IgG ELISA kit (InBios International, Inc. USA) following the manufacturer’s guidelines. Briefly, 50 μL of 1:100 prediluted serum and control were incubated in 96-well plates coated with monoclonal antibody bound to Dengue-derived recombinant antigen or bound to normal cell antigen for 1 h at 37 °C. The plate was washed and incubated with secondary anti-human IgG antibody conjugated with HRP for 1 h at 37 °C. The plate was washed again, and substrate TMB was added to the wells. After incubation at room temperature for 10 min, the stop solution was added to the wells. The OD 450 was determined, and the results were interpreted according to the guidelines. The assay was performed for each serum sample in duplicate. An acute-phase serum sample collected on days PSO ≤ 6 with a positive result was regarded as a recent secondary DENV infection. An acute-phase serum with a negative result was regarded as a recent primary DENV infection.

### DENV genomes and evolutionary analysis

We reconstructed the phylogenetic trees to investigate the possible origin of the DENV circulating in Taiwan during the 2015 dengue outbreak. The genomic sequences of the envelope protein E region of DENV-2 and DENV-1 generated from the clinical samples that were collected in Taiwan were retrieved and downloaded from GenBank. The sequences included in this study were uploaded by sequence authors between 2014 and 2019. There were no newly generated DENV-1 and DENV-2 sequences from the 2015 DF outbreak deposited in GenBank after 2019. Theoretical phylogenetic trees were reconstructed with the methods described in our previous studies [18, 19]. In brief, the sequences were aligned with MAFFT v 7.511 [20], the most appropriate evolutionary model used in the construction of the phylogenetic tree was evaluated, and theoretical phylogenetic trees were reconstructed with 1,000 bootstrap replicates by using IQ-TREE 2.2.0 [21]. The phylogenetic tree is displayed using FigTree v1.4.4 with bootstrap values and a scale bar.

### Clinical data collection and dengue case definitions

Demographic data and underlying chronic diseases were collected through electronic medical records. A DF case was defined as a patient associated with dengue-like illness and a positive DENV quantitative reverse transcription-polymerase chain reaction (qRT‒PCR) result, while those with a negative qRT‒PCR result were excluded from the study thereafter. A DF case was categorized as severe dengue fever when the patient had one of the following symptoms: 1. Severe plasma leakage leading to shock, 2. Severe plasma leakage leading to fluid accumulation with respiratory distress, 3. Severe bleeding, or 4. Severe impaired organ involvement, such as liver (AST or ALT ≥ 1,000), central nervous system (e.g., impaired consciousness), heart and other organs [2].

### Retrieval of dengue data in Taiwan

The epidemiologic dengue data in Taiwan were retrieved from the web-based notifiable diseases surveillance system (https://nidss.cdc.gov.tw/en/Home/Index) maintained by the Taiwan Centers for Disease Control (CDC). These data are publicly available. The imported cases and those that came from autochthonous transmission were the Taiwan CDC’s decision.

### Statistical analysis

Statistical analysis was performed using SPSS Statistics software version 19.0 (IBM Corp., USA). The independent samples T test (two-tailed) was used to analyze age, days PSO and Charlson comorbidity index (CCI) score, an assessment tool designed specifically to predict long-term mortality [22], between the different types of grouping. The significance difference between continuous variables was determined using the chi-square test. For those variables with significant differences in the univariate analysis, logistic regression was used to obtain odds ratios (ORs) and 95% confidence intervals (CIs) in the multivariate analysis. The results were considered statistically significant at P < 0.05.

## Results

### DENV-1 was detected in Kaohsiung city during the DF outbreak between September and December 2015

**Fig. 1:**
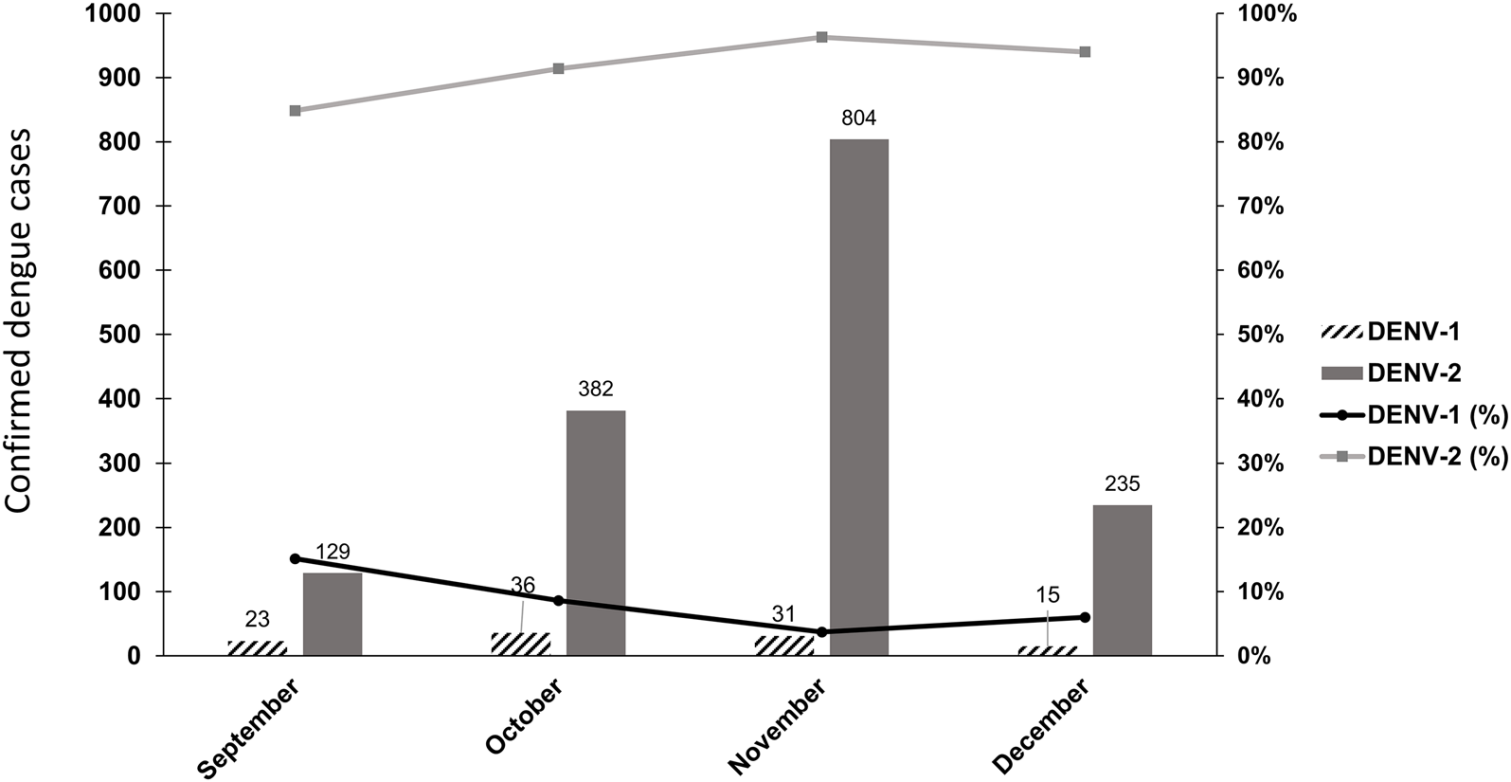
Monthly distribution of DF patients infected with DENV-1 or DENV-2 between September 2015 and December 2015 in Kaohsiung city

The largest DF outbreak in the past four decades in Taiwan occurred in 2015 (Additional file 1: Fig. S1), with a total of 43,784 DF cases and 224 deaths reported. Most DF cases were distributed in cities in southern Taiwan (Additional file 1: Fig. S2). We started to collect serum samples from hospitalized DF patients in September 2015, which was immediately after DENV-2 had spread from Tainan city to Kaohsiung city in August 2015 [9]. We collected a total of 1,655 single serum samples of DENV qRT‒PCR-positive patients, who were primarily adults (89.1% ≥ 20 y/o), between September and December 2015 in Kaohsiung city during the largest DF outbreak since World War II. The results of serotype-specific RT‒PCR revealed that 84.6–96.0% of the DF patients were infected by DENV-2, and 4.0–15.4% of the DF patients were infected by DENV-1 between September and December 2015 (Fig. 1). Our results suggested that most of the DF patients were infected by DENV-2, and DENV-1 still circulated in Kaohsiung city after the DF cases surged in September 2015. However, a previous study reported that DENV-1 was not detected after September 2015 [9]. Our results confirmed that DENV-2 was the major DENV in Kaohsiung city in the 2015 DF outbreak.

### Analysis results of the clinical effects of DENV serotypes and the risk factors for severe dengue and mortality

The demographic characteristics of the 1,655 DF patients enrolled in this study are shown in Table 1. There were more patients infected by DENV-2 (93.7%, n = 1,550) than those infected by DENV-1 (6.3%, n = 105). In the results of the multivariate analysis, there was no significant difference in a single parameter between the patients infected with DENV-1 and those with DENV-2. Thus, those who were infected with DENV-2 did not have a higher chance of developing severe dengue or a higher chance of death. The results were not consistent with the results that DENV-2 had a higher risk of DHF and severe dengue [11, 13]. Next, we analyzed whether any demographic characteristics, including age, sex, days post-infection, disease severity, comorbidities (e.g. diabetes mellitus, hypertension, congestive heart failure, chronic obstructive pulmonary disease, cerebrovascular disease, and moderate to severe chronic kidney disease), and clinical outcomes, influenced the development of severe dengue (Table 2). The results suggested that DENV-infected patients with DM (OR = 2.178, 95% CI 1.088–4.362) and moderate to severe CKD (OR = 5.806, CI 1.994–16.902) had a higher risk of developing severe dengue. Further analyses were performed to understand which parameter was correlated with and contributed to death after DENV infection (Table 3). The results suggested that the proportion of patients who died was significantly higher in those who developed severe dengue (OR = 115.713, CI 39.545–338.588) and in those who had a higher CCI score (OR = 1.686, CI 1.144–2.484) (Table 3).

**Table 1:** Analysis of all dengue patients with different DENV serotypes

Demographic Characteristics^a^	DENV-1	DENV-2	P value	Logistic regression	
	n = 105	n = 1550		P value	OR (95% CI)
Age (Years)	48 (3–88)	52 (0–91)	0.079	0.235	1.007
0–59	78 (74.3%)	975 (62.9%)	0.019	0.27	1.524 (0.721–3.223)
≥60	27 (25.7%)	575 (37.1%)			
0–9	4 (3.8%)	50 (3.2%)	0.011	0.104	
10–19	5 (4.8%)	121 (7.8%)			1.934 (0.499–7.503)
20–29	23 (21.9%)	170 (11.0%)			0.588 (0.194–1.780)
30–39	12 (11.4%)	242 (15.6%)			1.603 (0.496–5.179)
40–49	12 (11.4%)	145 (9.4%)			1.018 (0.312–3.317)
50–59	22 (21.0%)	247 (15.9%)			0.851 (0.265–2.735)
60–69	14 (13.3%)	273 (17.6%)			1.329 (0.353–5.002)
≥70	13 (12.4%)	302 (19.5%)			1.507 (0.294–7.741)
Sex					
Male	56 (53.3%)	792 (51.1%)	0.657		
Female	49 (46.7%)	758 (48.9%)			
Days PSO	2 (0–8)	2 (0–22)	0.879		
Disease severity					
Dengue fever	103 (98.1%)	1443 (93.1%)	0.046	0.223	3.486 (0.469–25.935)
Severe dengue	2 (1.9%)	107 (6.9%)			
Chronic disease^b^					
Diabetes mellitus	6 (5.9%)	163 (11.1%)	0.101		
Hypertension	15 (14.7%)	248 (16.8%)	0.575		
Congestive heart failure	3 (2.9%)	29 (2.0%)	0.502		
COPD	6 (5.9%)	66 (4.5%)	0.513		
Cerebrovascular disease	3 (2.9%)	65 (4.4%)	0.479		
Moderate to severe CKD^c^	2 (2.0%)	21 (1.4%)	0.664		
Charlson comorbidity index score^b^	1 (0–7)	1 (0–10)	0.031	0.426	1.088 (0.884–1.339)
Outcome^d^					
Survived	103 (98.1%)	1498 (96.9%)	0.487		
Death	2 (1.9%)	48 (3.1%)			

^a^Presented as the numbers (%) except for average age, days PSO, and CCI score, which are shown as the median (range)

^b^N = 1,574, DENV-1 = 102, DENV-2 = 1,472. *COPD* Chronic obstructive pulmonary disease, *CKD* Chronic kidney disease

^c^Moderate CKD: stage 3A & 3B (GFR 45–59 ml/min & 30–44 ml/min), Severe CKD: stage 4 & 5 (GFR 15–29 ml/min & less than 15 ml/min)

^d^There were four patients who did not die from dengue fever in the DENV-2 group and were excluded from the analysis

**Table 2:** Analysis of all dengue patients with different severities

Demographic Characteristics^a^	Dengue fever	Severe dengue	P value	Logistic regression	
	n = 1546	n = 109		P value	OR (95% CI)
Age (Years)	50 (0–91)	72 (16–91)	< 0.001	0.206	3.687 (0.488–27.845)
0–59	1025 (66.3%)	28 (25.7%)	< 0.001	0.12	1.931 (0.842–4.429)
≥60	521 (33.7%)	81 (74.3%)			
0–9	54 (3.5%)	0	< 0.001		
10–19	125 (8.1%)	1 (0.9%)			
20–29	193 (12.5%)	0			
30–39	246 (15.9%)	8 (7.3%)			
40–49	152 (9.8%)	5 (4.6%)			
50–59	255 (16.5%)	14 (12.9%)			
60–69	269 (17.4%)	18 (16.5%)			
≥ 70	252 (16.3%)	63 (57.8%)			
Sex					
Male	793 (51.3%)	55 (50.5%)	0.866		
Female	753 (48.7%)	54 (49.5%)			
Days PSO	2 (0–22)	2 (0–7)	0.755		
Serotype					
DENV-1	103 (6.7%)	2 (1.8%)	0.046	0.196	3.818 (0.501–29.118)
DENV-2	1443 (93.3%)	107 (98.2%)			
Chronic disease^b^					
Diabetes mellitus	143 (9.5%)	26 (39.4%)	< 0.001	0.028	2.178 (1.088–4.362)
Hypertension	234 (15.5%)	29 (43.9%)	< 0.001	0.607	1.178 (0.631–2.201)
Congestive heart failure	28 (1.9%)	4 (6.1%)	0.018	0.953	1.038 (0.308–3.499)
COPD	63 (4.2%)	9 (13.6%)	< 0.001	0.785	1.129 (0.473–2.695)
Cerebrovascular disease	63 (4.2%)	5 (7.6%)	0.184		
Moderate to severe CKD	15 (1.0%)	8 (12.1%)	< 0.001	0.001	5.806 (1.994–16.902)
Charlson comorbidity index score^b^	1 (0–10)	4 (0–8)	< 0.001	0.05	1.239 (1.000–1.534)
Outcome^c^					
Survived	1533 (99.3%)	68 (63.6%)	< 0.001		
Death	11 (0.7%)	39 (36.4%)			

^a^Presented as the numbers (%) except for average age, days PSO, and CCI score, which are shown as the median (range)

^b^N = 1,574, Dengue fever = 1,508, Severe dengue = 66. *COPD* Chronic obstructive pulmonary disease, *CKD* Chronic kidney disease

^c^There are two patients who did not die from either dengue fever or severe dengue

**Table 3:** Analysis of mortality in all dengue patients

Demographic Characteristics^a^	Survived	Death	P value	Logistic regression	
	n = 1601	n = 50		P value	OR (95% CI)
Age (Years)	50 (0–91)	74 (48–88)	< 0.001	0.167	1.044 (0.982–1.109)
0–59	1046 (65.3%)	7 (14.0%)	< 0.001	0.617	0.658 (0.128–3.392)
≥60	555 (34.7%)	43 (86.0%)			
0–9	54 (3.4%)	0	< 0.001		
10–19	126 (7.9%)	0			
20–29	193 (12.0%)	0			
30–39	254 (15.9%)	0			
40–49	156 (9.7%)	1 (2.0%)			
50–59	263 (16.4%)	6 (12.0%)			
60–69	281 (17.6%)	5 (10.0%)			
≥70	274 (17.1%)	38 (76.0%)			
Sex					
Male	827 (51.7%)	18 (36.0%)	0.029	0.065	2.560 (0.942–6.956)
Female	774 (48.3%)	32 (64.0%)			
Days PSO	2 (0–22)	2 (0–5)	0.717		
Serotype					
DENV-1	103 (6.4%)	2 (4.0%)	0.487		
DENV-2	1498 (93.6%)	48 (96.0%)			
Disease severity					
Dengue fever	1533 (95.8%)	11 (22.0%)	< 0.001	< 0.001	115.713 (39.545–338.588)
Severe dengue	68 (4.2%)	39 (78.0%)			
Chronic disease^b^					
Diabetes mellitus	151 (9.8%)	14 (45.2%)	< 0.001	0.961	1.032 (0.297–3.583)
Hypertension	247 (16.0%)	15 (48.4%)	< 0.001	0.431	0.639 (0.210–1.948)
Congestive heart failure	28 (1.8%)	4 (12.9%)	< 0.001	0.288	2.707 (0.431–17.015)
COPD	65 (4.2%)	6 (19.4%)	< 0.001	0.681	1.338 (0.334–5.352)
Cerebrovascular disease	64 (4.2%)	3 (9.7%)	0.132		
Moderate to severe CKD	20 (1.3%)	3 (9.7%)	< 0.001	0.453	0.517 (0.092–2.898)
Charlson comorbidity index score^b^	1 (0–10)	5 (1–8)	< 0.001	0.008	1.686 (1.144–2.484)

^a^Presented as the numbers (%) except for average age, days PSO, and CCI score, which are shown as the median (range)

^b^N = 1,570, survived = 1,539, death = 31. *COPD* Chronic obstructive pulmonary disease, *CKD* Chronic kidney disease

### Subanalysis of DENV-2-infected patients from the aspects of severity, mortality, and preexisting anti-dengue IgG

Since most patients were infected with DENV-2, we subanalyzed the characteristics of the hospitalized dengue patients infected with DENV-2 (n = 1,512). The results suggested that those who had a higher average age (OR = 1.049, CI 1.018–1.082), those who had DM (OR = 2.818, CI 1.382–5.748) or moderate to severe CKD (OR = 8.065, CI 2.676–24.308) had a significantly higher risk of developing severe dengue (Table 4). Next, we analyzed whether there were any demographic characteristics correlated with death from DENV-2 infection (Table 5). The results suggested that females (OR = 3.294, CI 1.145–9.474) and those with severe dengue (OR = 92.141, 30.775–275.874) had a significantly higher risk of death (Table 5). We detected preexisting anti-dengue IgG in a sex-matched panel of acute-phase sera from DENV-2-infected patients by using commercial ELISA. The results suggested that the presence of existing anti-dengue IgG in acutephase serum was correlated with severe dengue (P < 0.001, OR = 11.316, CI 3.051–41.967) and mortality (P = 0.004) (Additional file 1: Table S1).

**Table 4:** Subanalysis of the different severities in DENV-2-infected patients

Demographic Characteristics^a^	Dengue fever	Severe dengue	P value	Logistic regression	
	n = 1407	n = 105		P value	OR (95% CI)
Age (Years)	50 (0–91)	72 (16–91)	< 0.001	0.002	1.049 (1.018–1.082)
0–59	921 (65.5%)	26 (24.8%)	< 0.001	0.067	2.225 (0.947–5.228)
≥60	486 (34.5%)	79 (75.2%)			
Sex					
Male	719 (51.1%)	53 (50.5%)	0.902		
Female	688 (48.9%)	52 (49.5%)			
Chronic disease^b^					
Diabetes mellitus	136 (9.9%)	26 (40.6%)	< 0.001	0.004	2.818 (1.382–5.748)
Hypertension	216 (15.7%)	29 (45.3%)	< 0.001	0.735	1.114 (0.597–2.079)
Congestive heart failure	25 (1.8%)	4 (6.3%)	0.014	0.739	1.228 (0.367–4.114)
COPD	56 (4.1%)	9 (14.1%)	< 0.001	0.505	1.353 (0.556–3.291)
Cerebrovascular disease	59 (4.3%)	5 (7.8%)	0.183		
Moderate to severe CKD	13 (0.9%)	8 (12.5%)	< 0.001	< 0.001	8.065 (2.676–24.308)
Charlson comorbidity index score^b^	1 (0–10)	4.5 (0–8)	< 0.001	0.956	0.992 (0.760–1.297)
Outcome^c^			< 0.001		
Survived	1395 (99.3%)	65 (63.1%)			
Death	10 (0.7%)	38 (36.9%)			

^a^Presented as the numbers (%) except for average age and CCI score, which are shown as the median (range). The days PSO of these patients were ≤ 6 days

^b^N = 1,436, Dengue fever = 1,372, Severe dengue = 64. *COPD* Chronic obstructive pulmonary disease, *CKD* Chronic kidney disease

^c^There are two patients who did not die from dengue fever or severe dengue

**Table 5:** Subanalysis of mortality in DENV-2-infected patients

Demographic Characteristics^a^	Survived	Death	P value	Logistic regression	
	n = 1460	n = 48		P value	OR (95% CI)
Age (Years)	51 (0–91)	74 (48–88)	< 0.001	0.18	1.044 (0.980–1.113)
0–59	941 (64.5%)	6 (12.5%)	< 0.001	0.77	0.777 (0.144–4.202)
≥60	519 (35.5%)	42 (87.5%)			
Sex					
Male	752 (51.5%)	17 (35.4%)	0.028	0.027	3.294 (1.145–9.474)
Female	708 (48.5%)	31 (64.6%)			
Severity					
Dengue fever	1395 (95.5%)	10 (20.8%)	< 0.001	< 0.001	92.141 (30.775–275.874)
Severe dengue	65 (4.5%)	38 (79.2%)			
Chronic disease^b^					
Diabetes mellitus	144 (10.3%)	14 (46.7%)	< 0.001	0.661	1.331 (0.370–4.782)
Hypertension	229 (16.3%)	15 (50.0%)	< 0.001	0.323	0.563 (0.181–1.757)
Congestive heart failure	25 (1.8%)	4 (13.3%)	< 0.001	0.111	4.048 (0.725–22.607)
COPD	58 (4.1%)	6 (20.0%)	< 0.001	0.461	1.705 (0.413–7.042)
Cerebrovascular disease	60 (4.3%)	3 (10.0%)	0.131		
Moderate to severe CKD	18 (1.3%)	3 (10.0%)	< 0.001	0.796	0.789 (0.130–4.791)
Charlson comorbidity index score^b^	1 (0–10)	5 (1–8)	< 0.001	0.293	1.276 (0.810–2010)

^a^Presented as the numbers (%) except for average age and CCI score, which are shown as the median (range). The days PSO of these patients were ≤ 6 days. We excluded four patients from this analysis who did not die from severe dengue

^b^N = 1,432, survival = 1,402, death = 30. *COPD* Chronic obstructive pulmonary disease, *CKD* Chronic kidney disease

## Discussion

Two of the largest dengue outbreaks ever recorded in Taiwan occurred consecutively in 2014 (DENV-1) and 2015 (DENV-2). The number of confirmed DF cases in Kaohsiung city was 15,043 and 19,784 in 2014 and 2015, respectively, and these DF cases comprised 58.5% of all DF cases nationwide in these two years. Our results suggested that DENV-1 still circulated at 4.0–15.4% between September and December 2015 in Kaohsiung city after the number of DF cases surged in September 2015, which is in contrast to a previous study that reported that DENV-1 was not detected after September 2015 [9]. That study enrolled hospitalized dengue patients and performed RT-PCR for serotyping. Their methods were similar to those used in this study. The reason why they did not detect DENV-1 might be that the sample size was relatively low in their study (n = 300), so they might have missed DENV-1 in surveillance. The reason for this prolonged circulation of DENV-1 might have been the constant importation of DENV-1 from neighboring Southeast Asian countries [8, 10]. A recent study by Pan et al. revealed that 63.9% of imported cases had a concealment period of > 3 days in 2015. This led to the possibility that DENV-1 may have circulated in the community [10]. Although cocirculation of multiple serotypes of DENV increased the risk of being coinfected with more than one dengue serotype and was correlated with severe dengue, we did not find concurrent infection in the same individual by multiple dengue virus serotypes. In this study, we did not find any patients infected with DENV-3 or DENV-4. Previous results suggested that DENV-2 correlated with severe dengue and was associated with a higher proportion of patients with severe dengue than the other serotypes [11]. Lin et al. suggested a cosmopolitan genotype DENV-2 had high transmissibility to Aedes mosquitoes and was highly virulent in type I and type II interferon-deficient mice, with robust replication in spleen, lung, and intestine [23]. In contrast, Yung et al. demonstrated that DENV-1 infection may be more severe than DENV-2 infection [12]. However, in our study, there was no significant difference in any demographic characteristic between those who were infected with DENV-1 and DENV-2. We also found that those who were infected with DENV-2 did not have a higher risk of developing severe dengue, resulting in death, than those who were infected with DENV-1 and vice versa. The results in this study agree with our previous results that there is no significant difference between DENV-2 and other serotypes (e.g., DENV-3) in the development of severe dengue [24]. The dengue virus serotype (DENV-1 and DENV-2) did not contribute to clinical severity and mortality in Taiwan’s largest dengue outbreak in 2015. Our results were not consistent with previous results, which showed that DENV-2 had a higher risk of DHF and severe dengue [11, 13]. Our observations suggested that DENV-2 was not responsible for the relatively high mortality rate in 2015 compared with 2014. We found that people with DM and CKD had a significantly higher risk of developing severe dengue, and those with severe dengue or higher CCI scores had a significantly higher risk of death. Rowe et al. reported that elderly individuals (≥ 60) suffered more severe disease with more DHF (29.2% vs. 21.4%, P = 0.002) and severe dengue (20.3% vs. 14.6%, P = 0.006) than adults (< 60) [25]. Our results revealed that the elderly suffered more severe dengue, which was similar to their results (74.3% vs. 25.7%, P < 0.001). Our results also suggested that elderly patients accounted for the majority of dengue deaths (86.0% vs. 14.0%, p < 0.001), while their results suggested that dengue death between the elderly and the adults had no significant difference (P = 1). However, the reason for the difference between the two findings is not clear. The results of a recent meta-analysis by Chagas et al. suggested that DSS and DM are associated with mortality in patients with dengue [26]. Our results suggested that DM and severe dengue were associated with dengue death. The term "severe dengue" is used to describe the most serious forms of dengue disease, which can include both DHF and DSS. Thus, our results agreed with their findings. In addition, our results conformed to the conclusion in a study conducted in Thailand by Huang et al. that “our results suggest a continuing increase in age of DHF cases, shifting the burden toward individuals with more comorbidity” [27].

In the subanalysis of DENV-2-infected patients, the results revealed that patients who developed severe dengue had a higher average age than those who developed DF. Patients with DM or moderate to severe CKD were at higher risk of developing severe dengue after infection with DENV-2. Furthermore, the results suggested that females and those who develop severe dengue are at higher risk of death after infection with DENV-2. An earlier study reported that girls suffered from more severe illness than boys [28]. In addition, the results from the study by Anders et al. suggested that girls had a significantly higher risk of DSS and death [29]. Sex hormones delineate the distinctions between males and females, and the varying endocrine conditions could potentially impact both human physiology and pathophysiology. Sex-specific disparities can be discerned in numerous immune-related conditions, although many of these aspects have not yet undergone comprehensive evaluation [30]. The mechanism(s) of why females are at higher risk for severe dengue and dengue-related death is still unknown. In addition, the presence of anti-dengue IgG correlated with severe dengue and mortality. The results in this study are different from the peak dengue hospitalization in children or young adults, and severe dengue cases predominate and occur more often in children than in the elderly [29, 31, 32]. The results of preexisting anti-dengue IgG in the current study were consistent with our previous results that anti-DENV IgG seroprevalence rates increased with age, from 2% (20–29 y/o), 4.8% (30–39 y/o), 32.1% (60–69 y/o), and 62.9% (70–79 y/o) in healthy participants living in a highly epidemic district, Sanmin District, and seroprevalence rates were 4.8% (20–49 y/o), 9.3% (60–69 y/o) and 36.4% (70–79 y/o) in a low epidemic district, Nanzih District [33]. Recent studies suggested that severe dengue was characterized by elderly patient cases and multiple comorbidities in the 2015 dengue outbreak in Tainan city and Kaohsiung city [9, 34, 35]. Wang et al. reported that age ≤ 60 years, chronic diseases (hypertension and diabetes), preexisting anti-dengue IgG antibodies, thrombocytopenia, AST elevation, and APTT prolongation were correlated with DHF during the 2015 DF outbreak in Kaohsiung city [9]. The results by Chang et al. revealed that relatively high percentages of diabetes mellitus, hypertension, cerebral vascular accident, proton pump inhibitor use, severe hepatitis, and acute renal failure and relatively high maximum AST, maximum ALT, and initial white blood cells were observed in DHF patients in 2015 compared with 2014 [35]. Hsieh et al. reported that organ failure (cardiac failure and renal failure) and prolongation of initial APTT were predictive factors for in-hospital fatality during the 2015 DF outbreak in Tainan city. In addition, their data suggested that the proportion of severe dengue patients with hypertension, diabetes, and dyslipidemia was not significantly different between survivors and nonsurvivors [34]. However, their analysis did not mention which serotype of DENV the patients were infected with. Taken together, these results and our results indicate that dengue patients with chronic diseases, especially DM and moderate to severe CKD, have a higher risk of developing severe dengue. Our results also suggested that surveillance of the prevalent dengue virus serotype(s) can alert healthcare facilities to prepare for a potential increase in severe dengue cases and allocate resources accordingly. Testing for preexisting anti-dengue IgG (Additional file 1: Table S1) in the blood of dengue fever patients may provide an early warning of the development of severe dengue when a serotype is prevalent after other dengue virus serotypes have been circulating for many years. Public health agencies and researchers can prioritize investigations of prevalent serotypes to gain a deeper understanding of their genetic characteristics and virulence factors. Tailoring public awareness campaigns to highlight the major dengue serotypes can educate communities about the specific risks associated with prevalent serotypes, thereby promoting relevant preventive measures.

The severe dengue incidence was relatively high (6.6%) in our study when compared to nationwide data (1.49%) [7]. This could be because DF patients have relatively severe manifestations compared to overall community cases seeking medical treatment in medical centers. In contrast, the DF death rate was relatively low (2.4%) in our study when compared to nationwide data (5.15%) [7]. Our results revealed that aged patients and those who had DM or CKD were prone to severe dengue. Notably, the prevalence of DM and CKD increases with age in Taiwan [36, 37]. It is likely that the comorbidity instead of severe dengue alone makes the patients die. Other factors associated with dengue disease severity and clinical outcome include innate immunity [38], genetic background and the presence of secondary infections [39]. Recent study suggested mechanical transmission of DENV without an incubation period is expected to influence not only the timing, but also the severity, of DENV outbreaks [40]. These factors might play a role in the relatively high mortality rate in the 2015 DF outbreak in Taiwan. However, we did not include immunity and genetic background in our study. Primary DENV infection is characterized by the presence of anti-DENV IgM antibodies at 3 to 5 days PSO, and anti-DENV IgG antibodies usually become detectable at 7 to 10 days PSO. A low level of anti-DENV IgG antibodies persisted for decades after recovery from DENV infection and was an indication of a past DENV infection. During secondary DENV infection, anti-DENV IgG antibodies become detectable at high levels even in the initial phase of infection [2, 41–44]. It is suggested that heterotypic secondary infection might result in a more severe form of dengue, such as DHF. It is likely that the anti-DENV IgG detected as shown in Additional file 1: Table S1 was heterotypic to DENV-2 since the major DENV strains attributable to DF epidemics were DENV-1 and DENV-3 in Kaohsiung city between 2005 and 2014. Notably, the patients enrolled in this study were all hospitalized, so they were unlikely to be infected with the same serotype as the previous DENV infection. Thus, heterotypic secondary infection might play a role in the higher mortality rate in the 2015 dengue outbreak than in the 2014 outbreak. However, more studies need to be conducted to verify this inference.

**Fig. 2:**
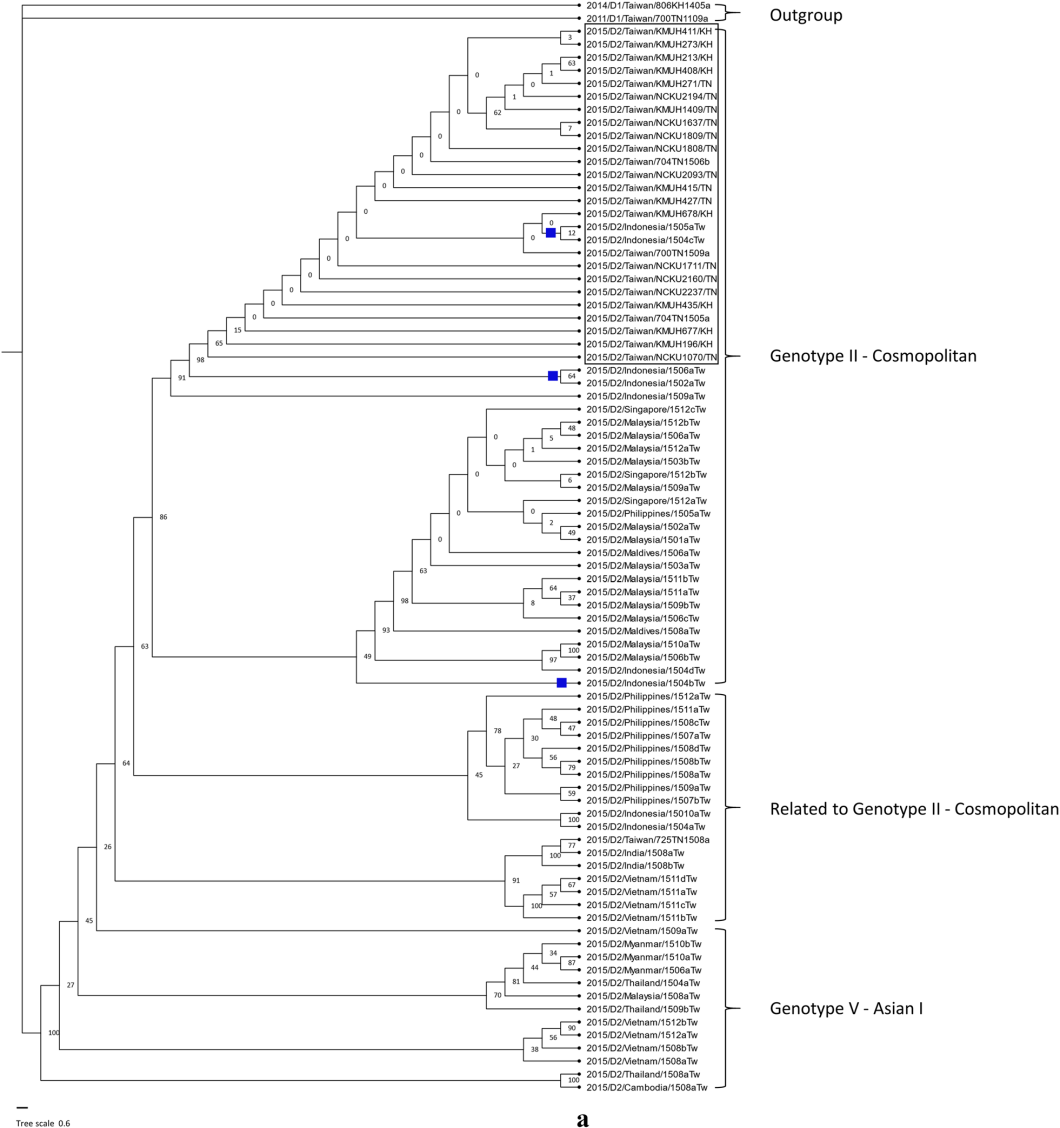

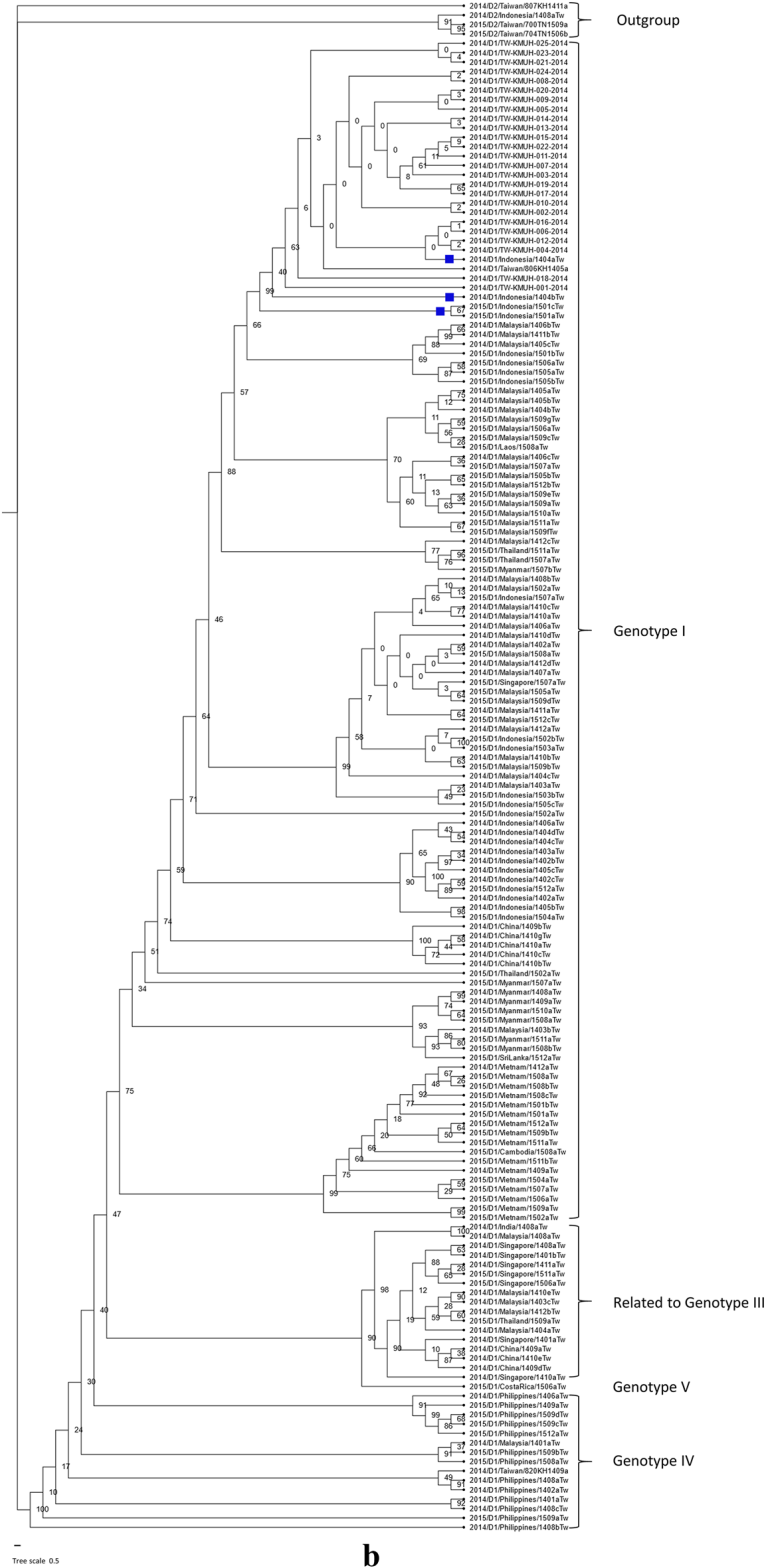
Phylogenetic tree of available genomic sequences of the DENV-2 and DENV-1 E genes, for which clinical samples were collected in Taiwan during the DF outbreak in 2015. **a** An original phylogenetic tree of 82 genomic sequences of the DENV-2 E gene obtained from GenBank. Two DENV-1 sequences were used as an outgroup. The DENV-2 sequences from which the clinical samples were collected from autochthonous cases were phylogenetically close to the sequences from the clinical samples that were collected from Indonesia. A virus with the abbreviation TN in its name was isolated from clinical samples collected in Tainan city. A virus with the abbreviation KH was isolated from clinical samples collected in Kaohsiung city. The two letters “Tw” at the end of the virus name mean that the sequence was imported into Taiwan from the country in the name. The phylogenetic analysis was inferred by using the maximum likelihood and fits of 484 different nucleotide substitution models, and the results suggested that TIM2 + F + I + G4 was the best-fitting model with the lowest Bayesian information criterion (BIC) scores of 14,173.117 among the 484 models tested. The tree topology was automatically computed to estimate maximum-likelihood values. The optimal log-likelihood for this computation was –6,612.703. There was a total of 1377 positions in the final dataset. Blue squares indicate cases from Indonesia whose evolutionary relationship is close to autochthonous Taiwanese DF cases. The box around a grouping of sequences highlights the autochthonous cases without travel history, except for the two cases from Indonesia with a small blue square on the left. **b** An original phylogenetic tree of 159 genomic sequences of the DENV-1 E gene obtained from GenBank. Four DENV-2 sequences were used as an outgroup. The phylogenetic analysis was inferred by using the maximum likelihood and fits of 484 different nucleotide substitution models, and the results suggested TN + F + I + I + R2 as the best-fitting model with the lowest BIC scores of 20,599.992 among the 484 models tested. The tree topology was automatically computed to estimate the maximum-likelihood values. The optimal log-likelihood for this computation was –9,229.897. There was a total of 1,485 positions in the final dataset. Blue squares indicate cases from Indonesia whose evolutionary relationship is close to autochthonous Taiwanese DF cases

Dengue is not endemic in Taiwan. Dengue outbreaks in Taiwan usually start with imported cases from Southeast Asian countries, spreading during rainy and warm weather starting in July and peaking between September and November [5]. Various serotypes and clades of DENV have been imported into Taiwan in the past twenty years from Southeast Asian countries, such as the Philippines, Thailand, Vietnam, Malaysia, Indonesia, Singapore, and Cambodia, and resulted in dengue epidemics of varying degrees [8, 45, 46]. It has been suggested that the business links and tourism between Taiwan and other Southeast Asian countries are responsible for the importation of multiple DENVs and dengue outbreaks in Taiwan [8]. To understand the possible origin of DENV-2 responsible for the DF outbreak in Taiwan in 2015, we retrieved genomic sequences of the DENV-2 E gene from GenBank to reconstruct the phylogenetic tree [47]. The clinical samples of these sequences were collected from dengue patients imported from abroad into Taiwan (virus name with the letters Tw) and from autochthonous DF patients in Taiwan (virus name with the letters TN or KH) in 2015 (Fig. 2a). Our results suggested that the DENV-2 circulated in Kaohsiung city (KH) was clustered with the DENV-2 circulated in Tainan city (TN), which was the genotype II – cosmopolitan clade – and possibly originated from Indonesia, while a previous report suggested that this DENV-2 is phylogenetically clustered with isolates from China and Thailand [7, 9]. Our results were consistent with the results from a previous study showing that the DENV-2 epidemic in Kaohsiung city was phylogenetically clustered with the DENV-2 epidemic in Tainan city [7]. In addition, most imported cases were from Indonesia, while the number of cases from Thailand and China was relatively low in 2015 (Additional file 1: Fig. S3). Furthermore, the possibility that the DENV-2 sequences included in the phylogenetic analysis in the previous studies [7, 9] were imported into Thailand and China from Indonesia had not been ruled out. Taken together, these results indicate that the DENV-2 in the 2015 DF outbreak in Taiwan possibly originated from Indonesia. On the other hand, DENV-1 sequences detected during the 2015 DF outbreak in Taiwan were imported from many Southeast Asian countries (Fig. 2b). Most of these sequences were phylogenetically close to the DENV-1 sequences detected in the dengue outbreak in 2014 from the same countries. In addition, our results confirmed that the 2014 epidemic DENV-1 in Taiwan originated from Indonesia [8].

There are strengths in our study. We have two novel findings from our study. First, we identified the serotypes of every enrolled dengue subject, which was not done in previous studies. Therefore, we found that DENV-1 circulation persisted in the community in addition to DENV-2 in the 2015 large outbreak. Second, we further found that severity and mortality were not associated with the dengue serotypes DENV-1 and DENV-2. Furthermore, we found that DENV-1 in 2015 evolved from that in 2014 from phylogenetic analysis.

There are some limitations to our study. First, we collected only single serum samples but not paired-serum samples in this study, so we could not perform anti-DENV antibody assays to include more potential DF cases using convalescence serum and follow-up on the symptoms, progression, and prognosis of dengue disease. Second, the sample size of DENV-1 is relatively low compared with that of DENV-2. Thus, we could not analyze the differences in clinical severity or clinical outcomes among hospitalized severe dengue patients infected with DENV-1. Third, the skewing of the data was due to collection only from hospitalized patients, which did not include many who would have been treated as outpatients and cases from the community. Those with milder disease or asymptomatic infections may have been missed. Due to these biases, our results reflected only those hospitalized patients and might not be representative of the overall outbreak in the community. Fourth, we reconstructed the phylogenetic trees using limited sequences and using the E gene but not the full-length DENV genome. Thus, the results in this study may not provide a complete picture of the evolutionary history of the dengue virus. Fifth, the dengue outbreak in Kaohsiung began in August 2015 and ended in March 2016 according to data from the Taiwan CDC. Although ~ 96% of the DF cases occurred during our study period, the timeframe did not capture the entirety of the outbreak. Thus, we might have missed earlier or later variations in serotype prevalence. Finally, the results of this cross-sectional study did not include factors such as urbanization, migration, changes in mosquito control strategies, climate change, and socioeconomic and ecological factors.

## Conclusions

Our results confirmed DENV-2 as the major serotype in the dengue outbreak in Kaohsiung city in 2015, while DENV-1 still circulated in the community. Dengue serotypes (DENV-1 and DENV-2) did not contribute to either severity or mortality. Aged patients and those who had DM and moderate to severe CKD were at a higher risk for severe dengue. Higher CCI scores and dengue severity cast a higher risk of mortality. For DENV-2-infected patients, we found that age, the presence of preexisting anti-dengue IgG, DM, and moderate to severe CKD were associated with a significant risk for severity, and female sex and severity contributed to death. Therefore, we should focus on the meticulous management of dengue with DM and moderate to severe CKD to lower the severity and prevent mortality. In addition, testing for preexisting anti-dengue IgG in dengue patients may provide early warning of the development of severe dengue when one serotype is circulating years after other dengue virus serotypes circulated.

### Supplementary Information

**Additional file 1: Table S1.** Subanalysis of preexisting anti-dengue IgG in DENV-2-infected patients. **Fig. S1.** Annual confirmed dengue fever cases between 1981 and 2022 in Taiwan. The dominant strain in the major outbreaks is shown above the bar. Source of data: Data from a previous study [48] and the Centers for Disease Control, Taiwan [49]. **Fig. S2.** Geographical distribution of confirmed dengue fever cases in Taiwan in 2015. Data on confirmed dengue case numbers were retrieved from the web-based notifiable diseases surveillance system maintained by the Centers for Disease Control, Taiwan [49] and are shown in parentheses in each second-level administrative division. This figure was generated using Quantum GIS v3.28.4 (QGIS Development Team, 2023. QGIS Geographic Information System. http://www.qgis.org/en/site/). Taiwan map data were retrieved from the Taiwan Geospatial One-Stop Portal developed by the Information Center of the Taiwan Ministry of The Interior and used under the pen Government Data License. The arrow points north. **Fig. S3.** Imported dengue fever cases in Taiwan in 2015. Most imported DF cases were from Southeast Asian countries. Data were retrieved from the web-based notifiable disease surveillance system maintained by the Centers for Disease Control, Taiwan [49]. Source of data: https://nidss.cdc.gov.tw/en/Home/Index.

## Data Availability

The datasets supporting the conclusions of this article are included within the article (and its additional files) and are available upon request.
