# Supplementary material for: Dengue virus serotype did not contribute to clinical severity or mortality in Taiwan’s largest dengue outbreak in 2015
Source: Eur J Med Res. 2023 Nov 6;28:482. doi: 10.1186/s40001-023-01454-3 (PMC10626727; doi:10.1186/s40001-023-01454-3)
Supplement: Supplementary file 1 — Additional file 1: Table S1. Subanalysis of preexisting anti-dengue IgG in DENV-2-infected patients. Fig. S1. Annual confirmed dengue fever cases between 1981 and 2022 in Taiwan. The dominant strain in the major outbreaks is shown above the bar. Source of data: Data from a previous study [48] and the Centers for Disease Control, Taiwan [49]. Fig. S2. Geographical distribution of confirmed dengue fever cases in Taiwan in 2015. Data on confirmed dengue case numbers were retrieved from the web-based notifiable diseases surveillance system maintained by the Centers for Disease Control, Taiwan [49] and are shown in parentheses in each second-level administrative division. This figure was generated using Quantum GIS v3.28.4 (QGIS Development Team, 2023. QGIS Geographic Information System. http://www.qgis.org/en/site/). Taiwan map data were retrieved from the Taiwan Geospatial One-Stop Portal developed by the Information Center of the Taiwan Ministry of The Interior and used under the pen Government Data License. The arrow points north. Fig. S3. Imported dengue fever cases in Taiwan in 2015. Most imported DF cases were from Southeast Asian countries. Data were retrieved from the web-based notifiable disease surveillance system maintained by the Centers for Disease Control, Taiwan [49]. Source of data: https://nidss.cdc.gov.tw/en/Home/Index. [file 40001_2023_1454_MOESM1_ESM.docx]

Additional file 1

**Dengue virus serotype did not contribute to clinical severity or mortality in Taiwan’s largest dengue outbreak in 2015**

Jih-Jin Tsai^1,2,3^, Ko Chang^1,2,3,4^, Chun-Hong Chen^5,6^, Ching-Len Liao^5,6^, Liang-Jen Chen^7^, Yan-Yi Tsai^1^, Ching-Yi Tsai^1^, Ping-Chang Lin^1^, Miao-Chen Hsu1, Li-Teh Liu^8^*

^1^ Tropical Medicine Center, Kaohsiung Medical University Hospital, Kaohsiung city, Taiwan,

^2^ School of Medicine, College of Medicine, Kaohsiung Medical University, Kaohsiung city, Taiwan,

^3^ Division of Infectious Diseases, Department of Internal Medicine, Kaohsiung Medical University Hospital, Kaohsiung city, Taiwan,

^4^ Department of Internal Medicine, Kaohsiung Municipal Siaogang Hospital, Kaohsiung Medical University, Kaohsiung city, Taiwan,

^5^ National Mosquito-Borne Diseases Control Research Center, National Health Research Institutes, Miaoli county, Taiwan,

^6^ National Institute of Infectious Diseases and Vaccinology, National Health Research Institutes, Miaoli county, Taiwan,

^7^ Department of Family Medicine, Pingtung Christian Hospital, Pingtung city, Taiwan,

^8^ Department of Medical Laboratory Science and Biotechnology, College of Medical Technology, Chung-Hwa University of Medical Technology, Tainan city, Taiwan.

*Correspondence: Li-Teh Liu; [liult0119@gmail.com](mailto:liult0119@gmail.com)

**Table S1 Subanalysis of preexisting anti-dengue IgG in DENV-2-infected patients**

| **Demographic**  **characteristics^a^** | **Dengue IgG negative**  **n=78** | **Dengue IgG positive**  **n=139** | ***P* value** | **Logistic regression** | |
| --- | --- | --- | --- | --- | --- |
|  |  |  |  | ***P* value** | **OR (95% CI)** |
| **Age** | 41.55 ± 20.67 | 64.04 ± 16.98 | <0.001 | 0.303 | 1.020 (0.982-1.060) |
| 0-59  ≥ 60 | 62 (79.5%)  16 (20.5%) | 41 (29.5%)  98 (70.5%) | <0.001 | 0.154 | 3.297 (0.640-16.986) |
| **Gender**  Male  Female | 41 (52.6%)  37 (47.4%) | 67 (48.2%)  72 (51.8%) | 0.537 |  |  |
| **Severity**  Dengue fever  Severe dengue | 68 (87.2%)  10 (12.8%) | 72 (51.8%)  67 (48.2%) | <0.001 | <0.001 | 11.316 (3.051-41.967) |
| **Outcome**  Survived  Death | 72 (92.3%)  6 (7.7%) | 107 (77.0%)  32 (23.0%) | 0.004 |  |  |

^a^ Presented as the numbers (%) except for average age, which is shown as the mean ± standard deviation. The days PSO of these patients were ≤ 6 days.


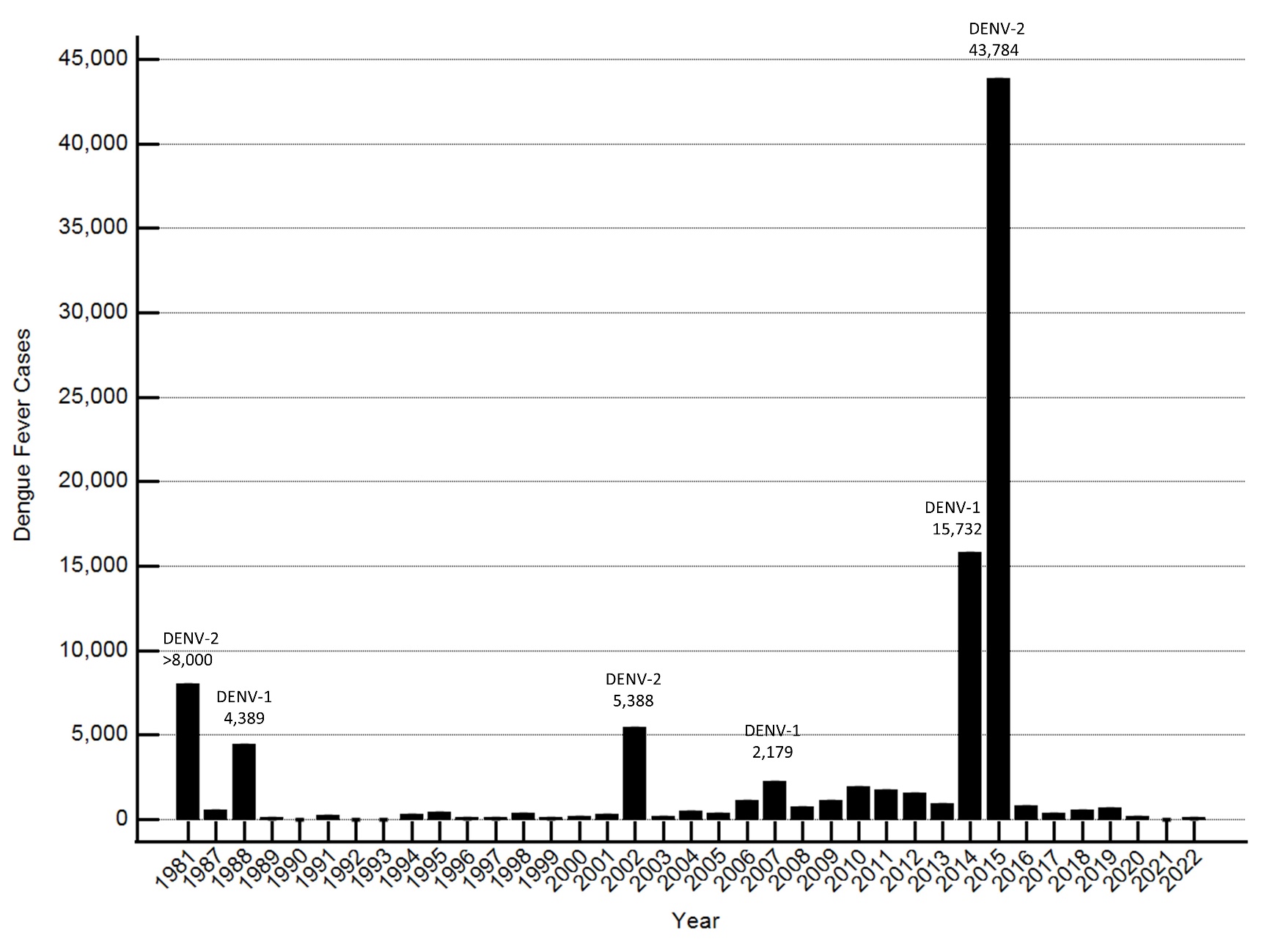
 **Fig. S1 Annual confirmed dengue fever cases between 1981 and 2022 in Taiwan**

The dominant strain in the major outbreaks is shown above the bar. Source of data: Data from a previous study [48] and the Centers for Disease Control, Taiwan [49].


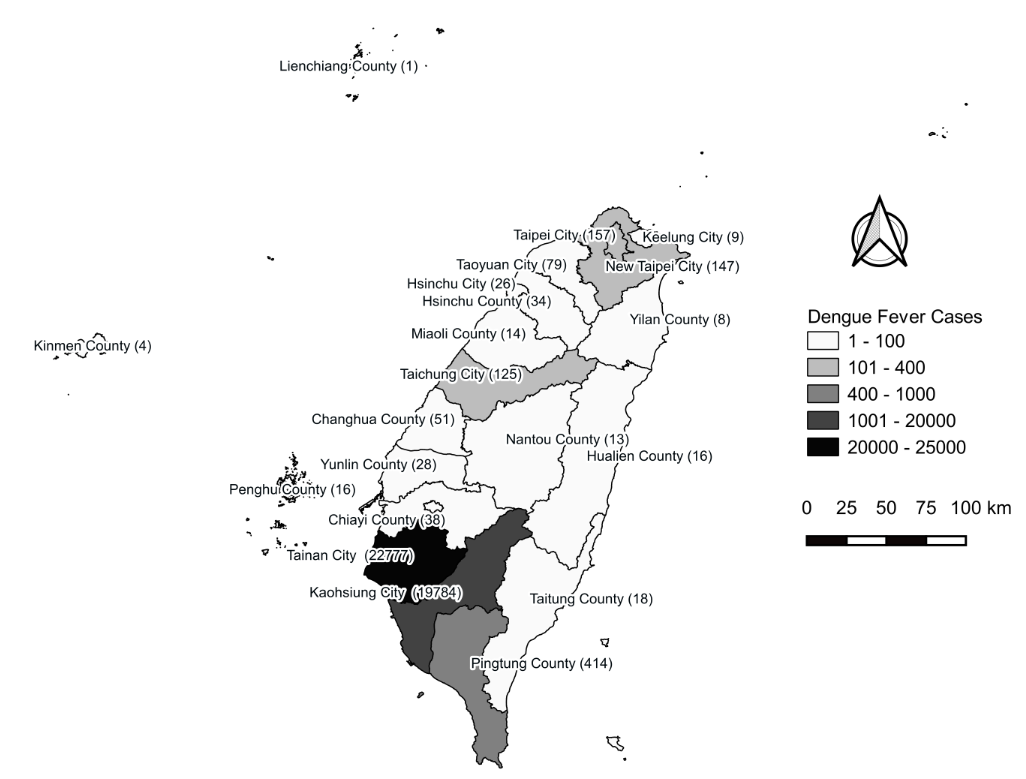
**Fig. S2 Geographical distribution of confirmed dengue fever cases in Taiwan in 2015**

Data on confirmed dengue case numbers were retrieved from the web-based notifiable diseases surveillance system maintained by the Centers for Disease Control, Taiwan [49] and are shown in parentheses in each second-level administrative division. This figure was generated using Quantum GIS v3.28.4 (QGIS Development Team, 2023. QGIS Geographic Information System. http://www.qgis.org/en/site/). Taiwan map data were retrieved from the Taiwan Geospatial One-Stop Portal developed by the Information Center of the Taiwan Ministry of The Interior and used under the pen Government Data License. The arrow points north.


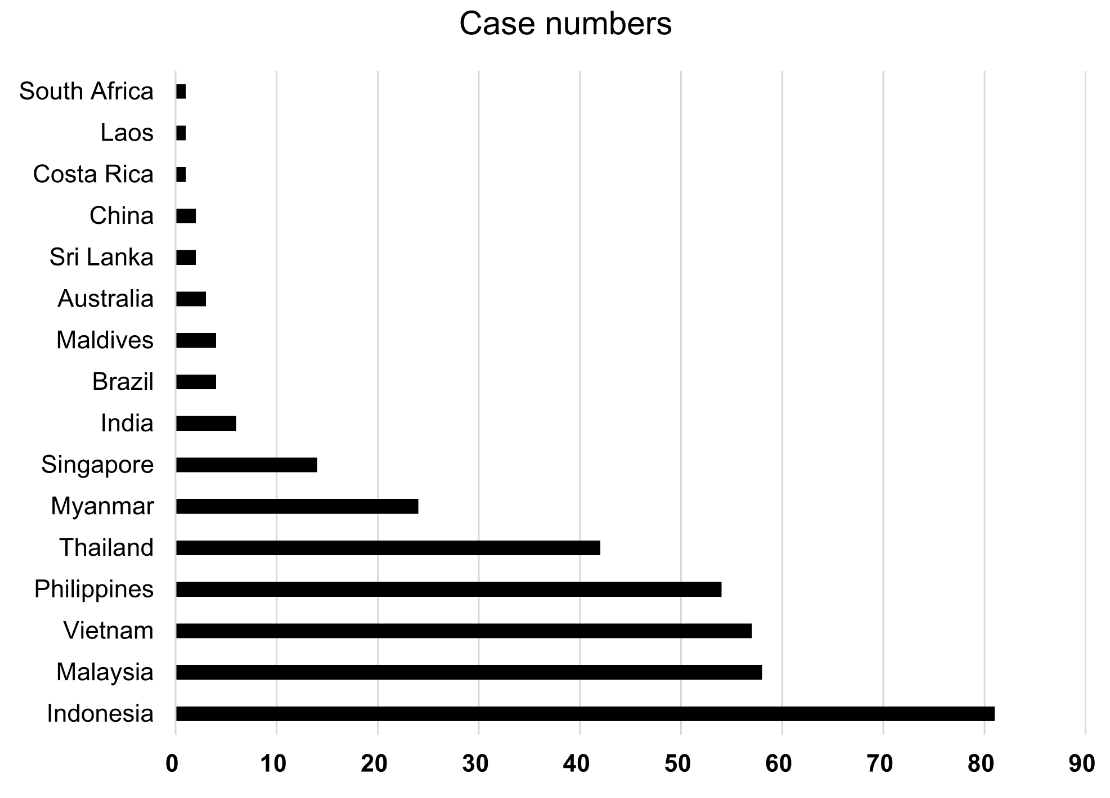
**Fig. S3 Imported dengue fever cases in Taiwan in 2015**

Most imported DF cases were from Southeast Asian countries. Data were retrieved from the web-based notifiable disease surveillance system maintained by the Centers for Disease Control, Taiwan [49]. Source of data: https://nidss.cdc.gov.tw/en/Home/Index.
